# Acceptance or Rejection of the COVID-19 Vaccine: A Study on Iranian People’s Opinions toward the COVID-19 Vaccine

**DOI:** 10.3390/vaccines10050670

**Published:** 2022-04-23

**Authors:** Amin Nakhostin-Ansari, Gregory D. Zimet, Mohammad Saeid Khonji, Faezeh Aghajani, Azin Teymourzadeh, Amir Ali Rastegar Kazerooni, Pendar Pirayandeh, Reyhaneh Aghajani, Sepideh Safari, Kamand Khalaj, Amir Hossein Memari

**Affiliations:** 1 Sports Medicine Research Center, Neuroscience Institute, Tehran University of Medical Sciences, Tehran 1417613151, Iran; a-nansari@alumnus.tums.ac.ir; 2 Department of Pediatrics, Indiana University School of Medicine, Indianapolis, IN 46202, USA; gzimet@iu.edu; 3 School of Medicine, Tehran University of Medical Sciences, Tehran 1417613151, Iran; ms-khonji@student.tums.ac.ir (M.S.K.); azin.teymourzadeh@gmail.com (A.T.); reyhaneh.aghajani1376@gmail.com (R.A.); kamand.kh17@gmail.com (K.K.); 4 Research Development Center, Arash Women’s Hospital, Tehran University of Medical Sciences, Tehran 1417613151, Iran; faezehaghajani.fa@gmail.com; 5 Student Research Committee, Shiraz University of Medical Sciences, Shiraz 7134814336, Iran; amirali.rastegar1376@gmail.com; 6 School of Medicine, Tonekabon Islamic Azad University, Mazandaran 4684161167, Iran; ppirayandeh@gmail.com; 7 School of Medicine, Islamic Azad University, Tehran 1949635881, Iran; sepiidesafarii@gmail.com

**Keywords:** COVID-19, vaccine, attitude, safety

## Abstract

We aimed to assess the Iranian people’s attitude and confidence in the COVID-19 vaccine, their concerns about the safety of vaccines, and their reasons for accepting or rejecting the COVID-19 vaccine. We conducted a web-based cross-sectional study with a mixed qualitative–quantitative approach from December 2020 to February 2021. Our questionnaire consisted of a COVID-19 vaccine attitude questionnaire, a COVID-19 vaccine confidence inventory, a modified vaccine safety scale, and questions about participants’ decision to accept or refuse the COVID-19 vaccine, and their explanations for their decisions. The research included 1928 people with an average age of 34.88 years with 1236 (64.1%) being female. A total of 1330 participants desired to have the COVID-19 vaccine (69%). Female gender, lower educational levels, following COVID-19 news through sources other than websites and social media, not following COVID-19 news, and loss of a first-degree relative due to COVID-19 were all associated with a more negative attitude toward the COVID-19 vaccine (*p* < 0.01). To conclude, the acceptance rate of the COVID-19 vaccine among Iranians is comparable to the international average; however, it is still unfavorable. There are serious challenges to the vaccination program in Iran, such as older adults’ lower intention to undergo the COVID-19 vaccine and misinformation.

## 1. Introduction

Given the extremely infectious nature of the virus that causes coronavirus disease 2019 (COVID-19), protective measures, including using face masks, social distancing, and disinfecting hands and surfaces, have been the main strategies to combat the pandemic [1,2]. Since the beginning of the pandemic, there have been intensive efforts to rapidly develop safe and efficacious vaccines against severe acute respiratory syndrome coronavirus 2 (SARS-CoV-2) [3,4,5]. Polack et al. announced in December 2020 that phase 3 clinical trials supported the efficacy and safety of an mRNA COVID-19 vaccine [5], and several countries have since planned for or initiated vaccination programs [6,7,8]. In order to achieve herd immunity against COVID-19, it is estimated that at least 67% of people in a fully susceptible population should be vaccinated [9], though the percentage may need to be substantially higher depending on the spread of viral variants.

Vaccine acceptance has historically been a challenging concept, and the COVID-19 vaccine is not an exception [10,11]. Several studies were conducted on the acceptability of the COVID-19 vaccine among different groups of people, including healthcare workers and the general population. The general population’s acceptance of the COVID-19 vaccine varies considerably across countries, ranging from 23.6% in Kuwait to 97% in Ecuador [12]. People’s attitudes toward the vaccine, their concerns regarding its safety, and their confidence in the vaccine are all major contributing factors to vaccine acceptance [13]. COVID-19 vaccine acceptability can shift over time as well, depending on reports of adverse events, political factors, and other issues.

In Iran, various measures were taken by the authorities to control the COVID-19 outbreak. These measures include partial lockdowns, remote working, distant education, and stopping mass gatherings [14]. These preventive measures, which have led to the closure of some industries, in addition to the medical costs of COVID-19, have imposed serious economic challenges on Iran’s economy [15,16]. Vaccination against COVID-19 was started in Iran in February 2021 as a more effective strategy to control the COVID-19 outbreak. Vaccination of high-risk populations, including healthcare workers, against COVID-19, was started at this time after the authorization of the Sputnik V vaccine. In the later stages, Sinopharm, AstraZeneca, and a number of homegrown vaccines against COVID-19, such as COVIran Barekat, were also authorized, and Sinopharm has been the most widely used vaccine against COVID-19 in Iran [17]. However, no study has evaluated the general populations’ attitude toward the COVID-19 vaccine and their decision to accept or reject the COVID-19 vaccine, despite its importance in vaccination programs. This issue is particularly salient considering the lack of sufficient knowledge and low intention of Iranian people to receive vaccines, which was demonstrated in previous studies [18,19,20]. Therefore, we aimed to assess the Iranian people’s attitudes toward the COVID-19 vaccine, their confidence in the COVID-19 vaccine, and their concerns about vaccines safety, just prior to the implementation of vaccination programs in Iran. Additionally, we aimed to investigate the Iranian people’s reasons to accept or reject the COVID-19 vaccine.

## 2. Methods

### 2.1. Design

After phase III clinical trials confirmed the safety and efficacy of some COVID-19 vaccines, and the United States (US) Food and Drug Administration (FDA) authorized the emergency use of the Pfizer-BioNTech and Moderna COVID-19 vaccines, the current study was conducted as a cross-sectional study from 20 December 2020 to 12 February 2021 [3,5,21,22]. In the current study, a mixed, quantitative–qualitative method was used. We used a convenience sampling method in our study. First, we identified popular channels and groups with more than 100,000 members on virtual platforms such as WhatsApp and Telegram. After that, we sent invitations to the groups and channels whose admins cooperated with us to do so and included an explanation of the study goals and objectives. We also mentioned that participation in the study was entirely voluntary, and responses would be anonymous. Those who did not want to take part in the study had the option to ignore the message. This study was conducted according to the declaration of Helsinki, and Tehran University of Medical Sciences’ institutional review board (IRB) approved the study protocol (Code: IR.TUMS.NI.REC.1400.062). The IRB waived parental consent for those who were younger than 18 years old as we wanted to include high school students who are mostly 15 to 18 years old in our study, and obtaining parental consent was not possible in an online survey.

### 2.2. Participants

Our inclusion criteria were as follows: 1. Age of 15 years or older, 2. Living in Iran during the COVID-19 pandemic, 3. Being Iranian, 4. Being able to read Farsi, and 5. Giving consent to participate in the study.

### 2.3. Measurements

The questionnaire assessed demographic characteristics, attitudes toward the COVID-19 vaccine, concerns about the safety of vaccines, confidence in the COVID-19 vaccine, and reasons for receiving or not receiving it. The scales were modified or created by an expert panel of three general practitioners.

We modified the Busse et al. vaccine attitude questionnaire [23] to create the questionnaire for evaluation of people’s attitudes toward the COVID-19 vaccine. The original questionnaire consisted of 11 items, but four were removed after the review by the expert panel, and the remaining items were modified to adapt to the COVID-19 vaccine. Each item could be answered with “agree”, “disagree”, and “neither agree nor disagree”. Each was scored from 0 to 2 with two points assigned to the most positive attitude, and no points assigned to the most negative attitude. The total score was the sum of the scores for all items and ranged from 0 to 14.

We modified the Rossen et al. vaccine confidence inventory to create a questionnaire to evaluate people’s degree of lack of confidence in the COVID-19 vaccine [13]. The original questionnaire consisted of 18 items, but three were excluded after review by the expert panel. The expert panel also modified the other items to make them compatible with the COVID-19 vaccine. Each item could be answered on a Likert scale ranging from 1 (strongly disagree) to 5 (strongly agree). Higher scores indicated less confidence in the COVID-19 vaccine, except in two items in which the scoring was reversed. The total score was the sum of the scores of all items and ranged from 15 to 75, again with higher scores indicating lower confidence in the COVID-19 vaccine.

The vaccine safety concerns scale [13] developed by Rossen et al. was modified to create a questionnaire to assess people’s concerns about vaccines in general. As there is still a need for more studies on possible side effects of COVID-19 vaccines, we used the general items of this questionnaire to evaluate the participants’ concerns regarding the safety of vaccines in general. The original questionnaire had 14 items; however, the expert panel kept six of them while excluding others. Each item could be answered on a Likert scale ranging from 1 (strongly disagree) to 5 (strongly agree), with higher scores indicating greater concerns about the safety of the vaccines. The total score was calculated as the sum across all items, with potential scores ranging from 6 to 30. After the expert panel approved the questionnaires, we distributed them to 30 people to evaluate the fluency of the sentences and to correct the sentences that were difficult to understand. After that, we sent the questionnaire to 100 people to evaluate its internal consistency, and once we determined that it was reliable, we approved it for use in our study. Supplementary S4 contains the final versions of the questionnaires.

Based on the total sample of participants, Cronbach’s alpha values for the COVID-19 vaccine attitude questionnaire, COVID-19 vaccine lack of confidence inventory, and modified vaccine safety scale were 0.83, 0.9, and 0.93, respectively, demonstrating good internal reliability. Following administration of these scales, we asked the participants whether they would receive the COVID-19 vaccine if it was available.

Furthermore, we asked individuals about their source of COVID-19 news and the time they spent reading COVID-19 news on a daily basis. Participants were asked whether they or a first-degree relative had been infected or hospitalized due to COVID-19. They were also asked if any of their first-degree relatives died of COVID-19.

### 2.4. Qualitative Part

As this study was conducted in the initial stages of vaccination programs and little was known about the factors leading to acceptance or rejection of the COVID-19 vaccine, we included an open-ended question at the end of our questionnaire in order to further investigate the reasons behind people’s decisions to accept or refuse the COVID-19 vaccine. As mentioned, it was an open-ended question (“Please explain your reasons for receiving or not receiving the COVID-19 vaccine”) and was included at the end of our questionnaire. All participants were invited to answer this question, and they could freely share their personal opinion anonymously. We utilized thematic qualitative analysis in order to identify common themes in answers to this question [24]. The two authors (ANA and AARK) who conducted this analysis are experienced in qualitative data analysis and in medical and public health research. These authors performed the data analysis independently and discussed disagreements to provide the final themes. All authors read and approved the final themes, subthemes, and codes.

### 2.5. Data Analysis

We calculated the number and percentage for categorical variables and the mean and standard deviation (SD) for continuous variables. We used the Kolmogorov–Smirnov test to evaluate whether the COVID-19 vaccine attitude questionnaire, COVID-19 vaccine confidence inventory and modified vaccine safety concerns scale scores were normally distributed. Because none of the questionnaires were distributed normally (*p* < 0.001), we used non-parametric tests, such as Mann–Whitney tests and Kruskal–Wallis analysis, to evaluate the differences in questionnaire scores between groups. Whenever we found significant differences between more than two groups, we used post-hoc analysis to evaluate the differences between pairs of groups. The factors independently associated with the COVID-19 vaccine attitude questionnaire, COVID-19 vaccine confidence inventory, and modified vaccine safety concerns scale scores were determined using multiple stepwise linear regression models. The Chi-square test was used to evaluate the willingness of different groups to receive the COVID-19 vaccine. *p* ≤ 0.05 was considered significant. For data analysis, we used SPSS software, version 22 (SPSS, Inc., Chicago, IL, USA).

## 3. Results

In total, 4604 people responded to our invitation to participate in the study, and of them, 1928 people from all 31 of Iran’s provinces took part in the study (response rate = 41.87%). The mean age of the participants was 34.88 years (SD = 12.74), ranging from 15 to 99 years. The majority of participants were female (*n* = 1236; 64.1%), while 692 (35.9%) were male. Most participants had a history of COVID-19 infection in themselves or their first-degree relatives (*n* = 1163; 60.3%), which resulted in hospitalization in 401 of them (20.8%). Demographic characteristics of participants as well as their history of COVID-19 infection are shown in Table 1.

### 3.1. Attitude toward COVID-19 Vaccine

The mean score on the COVID-19 vaccine attitude questionnaire was 9.94 (SD = 3.55). Participants showed the most negative attitudes toward the perceived lack of safety data (Supplementary S1). There was no association between participants’ residency areas (*p* = 0.698), age (*p* = 0.104), and history of COVID-19 infection in themselves or their first-degree relatives (*p* = 0.268) and their attitude toward the COVID-19 vaccine. There was a significant association between people’s attitudes toward the COVID-19 vaccine and other variables, which are presented in Table 2.

Following multiple linear regressions (Table 3), we found that female gender (beta = −0.531, *p* = 0.001), lower educational levels (beta = −1.33, *p* = 0.002), not following COVID-19 news during the day (beta= −1.615, *p* < 0.001), and death of first-degree relatives due to COVID-19 (beta = 0.849, *p* = 0.001) were associated with more negative attitudes toward the COVID-19 vaccine. On the other hand, a more optimistic attitude toward the COVID-19 vaccine was associated with higher educational levels (beta = 0.814, *p* < 0.001) and using websites and social media as the main source of COVID-19 news (beta = 1.213, *p* < 0.001).

### 3.2. Lack of Confidence in the COVID-19 Vaccine

The mean score on the COVID-19 vaccine lack-of-confidence inventory was 40.58 (SD = 10.87). Most people believed that the side effects of the COVID-19 vaccine are not well studied, and it is important to allow people to choose whether or not to receive the COVID-19 vaccine (Supplementary S2). There was no significant association between participants’ residency area (*p* = 0.301) and history of COVID-19 infection in themselves or their first-degree relative (*p* = 0.67). The associations between participants’ confidence in the COVID-19 vaccine and other variables, which were statistically significant, are shown in Table 2.

After multiple linear regression (Table 3), we found that single marital status (beta = −1.636, *p* = 0.002), having a master’s degree or higher (beta = −3.212, *p* < 0.001), being a student (beta = −2.132, *p* = 0.001), and using websites and social media as the main source of COVID-19 news (beta = −3.959, *p* < 0.001) are independently associated with greater COVID-19 vaccine confidence, as indicated by lower scores on the lack of confidence inventory. On the other hand, not following COVID-19 news throughout the day (beta = 5.222, *p* < 0.001) and the death of one’s first-degree relatives because of COVID-19 (beta = 2.032, *p* = 0.008) were associated with less confidence in the COVID-19 vaccine.

### 3.3. Concerns about the Vaccines’ Safety

The mean modified vaccine safety concern scale score was 15.76 (SD = 4.61). Participants were mainly concerned with the vaccines causing Autism Spectrum Disorders (ASD) in children and sudden infant death (Supplementary S3). Modified vaccine safety concern scale scores differed dramatically across genders, marital statuses, educational levels, occupations, incomes, and news sources (*p* < 0.05). However, there was no association between participants’ concerns regarding vaccines’ safety and their area of residency (*p* = 0.301), and history of COVID-19 infection in themselves or their first-degree relatives (*p* = 0.67). Associations between participants’ concerns regarding vaccines safety and variables are shown in Table 2.

Multiple linear regression analyses (Table 3) indicated that females (beta = 0.569, *p* = 0.008) and those who did not follow the COVID-19 news (beta= 1.42, *p* < 0.001) were more concerned about the safety of the vaccines. Additionally, an educational degree of masters or higher (beta = −0.831, *p* < 0.001), following COVID-19 news via websites and social media (beta = −1.397, *p* < 0.001), and being a healthcare worker (beta = −0.987, *p* = 0.01) were independently associated with fewer concerns about the COVID-19 vaccine.

### 3.4. Desire to Receive the COVID-19 Vaccine

In our survey, 1330 (69%) participants desired to receive the COVID-19 vaccine. We found that younger adults, males, single adults, and students had a greater intent to receive vaccination than other groups (*p* < 0.05). People who followed the COVID-19 news on social media or followed the COVID-19 for a longer period of time were more likely to choose to have the COVID-19 vaccine (*p* < 0.05). People who did not wish to pursue vaccination showed more concerns regarding the COVID-19 vaccine, were less confident about it and had a more negative attitude toward the vaccine (*p* < 0.001). The association between people’s preference to receive the COVID-19 vaccine and basic characteristics, their attitude, confidence, and concerns about the COVID-19 vaccine are all summarized in Table 4.

### 3.5. Reasons for Acceptance of the COVID-19 Vaccine (Qualitative Results)

One of the general findings was that some people mentioned that their decision to accept or reject the COVID-19 vaccine was dependent on the country that manufactured it. The reasons given by participants for receiving the COVID-19 vaccine could be categorized into six themes; ending the COVID-19 pandemic, responsibility, becoming immune against COVID-19, psychological well-being, hopefulness regarding vaccine effectiveness, and trust in the COVID-19 vaccine (Table 5).

#### 3.5.1. Ending the COVID-19 Pandemic

We found three subthemes related to this theme. One of the participants’ motives for COVID-19 vaccination was to control the COVID-19 pandemic by mass vaccination, leading to decreased transmission and herd immunity. According to one participant:

*“I think mass vaccination would be the only way to get rid of the current situation. Studies have shown that being infected by COVID-19 does not result in permanent immunity, and the pandemic will last until we get vaccinated.”* Female, 29 years old.

Getting tired of the current situation was another reason for receiving the COVID-19 vaccine. Some participants mentioned that they have gotten tired of being quarantined and taking health precautions; therefore, they would rather receive the COVID-19 vaccine.

*“I want to put aside the facemasks, antiseptics, and gloves.”* Female, 25 years old.

Return to the pre-pandemic life was another explanation for receiving the COVID-19 vaccine, as people wanted a normal situation in their social relationships and careers. One of the participants said:

*“My family’s health is important for me. Therefore, despite the fact that sports are one of my essential needs in life, I have canceled all of my athletic events even though it is one of my needs in life. After getting vaccinated, I can return to the normal situation with a bit of flexibility.”* Female, 28 years old.

She continued:

*“I am a student. I need to get back to the normal situation in order to continue my researchs. I’d like to attend university and participate in the in-person classes.”*

#### 3.5.2. Responsibility

We found three subthemes relate to this theme. A number of participants expressed a sense of community responsibility to encourage other people to be vaccinated and prevent further SARS-CoV-2 mutations. One participant said:

*“I get the COVID-19 vaccine because I am responsible for other people’s health.”* Female, 35 years old.

Another participant said:

“*SARS-CoV-2 is a viral disease with a high potential for novel mutations, leading to more devastating complications. So, mass vaccination is required to prevent novel mutations to occur*.” Female, 24 years old.

Another justification for certain individuals to have the vaccine was to protect those who are vulnerable to the disease or who are unable to receive the vaccine.

*“If young people like me have the COVID-19 vaccine, the transmission will slow down, and elderly people, children, and those who are allergic to vaccine compounds will be protected.”* Male, 20 years old.

According to other participants:

*“If I receive the COVID-19 vaccine, I will not become infected, and those who do not have access to or cannot afford the vaccine will become immune*.” Male, 55 years old *“We should think of those who may lose their life due to COVID-19 and reduce the risk for those high-risk populations.”* Male, 40 years old.

One of the participants said:

*“I lost my father because of COVID-19. I want to protect my mother.”* Male, 54 years old.

The main motivation of some people to receive the COVID-19 vaccine was to help the Health Care Workers (HCWs) and scientists:

*“If my vaccination against COVID-19 can help the medical sciences, even if it means losing my life, I am one hundred percent willing to help.”* Male, 58 years old.

#### 3.5.3. Becoming Immune against COVID-19

We discovered three subthemes connected to this theme. Some subjects felt it was important that they and their families be immune to the COVID-19. Avoidance of becoming the carrier of the COVID-19 was another code related to this subtheme.

One of the participants said:

*“I have taken health measures up to now, and I have not contracted COVID-19. “However, if the vaccine is shown to be effective, I would get it in order to reduce the risk of getting COVID-19 to zero.”* Male, 29 years old.

The primary reason for some participants receiving the COVID-19 vaccine was to prevent progression to severe forms of the disease. According to one of them:

“*COVID-19 is a dangerous disease, leading to irreversible physical and psychological complications, even after recovery.”* *Therefore I would receive the vaccine*.” Female, 21 years old.

Any participants believed they were vulnerable to COVID-19 whether they had high exposure to it often, had an underlying disease, or had a history of contracting COVID-19.

*“I am a nursing student, and I have strong exposure to the COVID-19. I prefer to receive the vaccine.”* Female, 22 years old.

#### 3.5.4. Psychological Well-Being

In this theme, we found two subthemes. Some participants were afraid of COVID-19 for different reasons, so they opted to have the COVID-19 vaccine. One of the participants said:

*“I will receive the COVID-19 vaccine, so I will not be concerned about being the carrier and transmitting COVID-19 to my family, and I will not be worried about losing my family.”* Female, 20 years old.

Alleviation of the psychological issues was the main reason for some participants:

*“The stress has impaired my job and life.”* Female, 27 years old.

#### 3.5.5. Hopefulness about Vaccine Effectiveness

We found two subthemes in this theme. Several respondents believed that receiving the COVID-19 vaccine was more cost-effective compared to other alternatives:

*“I am not sure of the safety of the COVID-19 vaccine because it has received emergency approval.”* One participant said: “However, *I think its advantages outweighs its drawbacks*.” Female, 21 years old.

Some participants wanted to take any action against COVID-19, even if it meant taking a risk:

*“It is like a shot in the dark.”* Male, 19 years old.

#### 3.5.6. Trust in the COVID-19 Vaccine

In this theme, we found two subthemes. Some participants believed the scientific evidence provided by scientists and experts regarding the COVID-19 vaccine.

“*Modern laboratories, pharmaceutical companies, and scientists have worked constantly to produce the safest vaccine*.” a participant said. “*Making COVID-19 vaccine in less than a year is a miracle in medicine, and we should respect them all as they save people’s lives*.” Male, 33 years old.

Some people trusted the COVID-19 vaccine because of previous successful vaccination programs in other countries.

“*I do not know anything about medicine”, one participant said, “but I’m sure the COVID-19 vaccine will be effective, just like other vaccines made for contagious diseases such as tetanus, measles, and rubella*.” Female, 47 years old.

### 3.6. Reasons for Rejection of the COVID-19 Vaccine (Qualitative Results)

Participants’ reasons to refuse the COVID-19 vaccine could be divided into six categories: 1–Unknown policies underlying the COVID-19 pandemic, 2–A lack of knowledge about the COVID-19 vaccine, 3–A lack of trust in the COVID-19 vaccine, 4–Responsibility, 5–Feeling no need for COVID-19 vaccine, and 6–Personal issues.

#### 3.6.1. Unknown Policies Underlying the COVID-19 Pandemic

We found three subthemes related to this theme. Some people believed that SARS-CoV-2 was purposefully spread throughout the world in order to use its vaccine for purposes such as harming people, controlling population growth, and controlling the world. According to one of the participants:

“*The plan to spread the virus throughout the world is used by dark governments to perform mass vaccination, implant nanochips into people’s bodies, and control the people*.” Female, 50 years.

“*COVID-19 is a political game”, said another participant, “vaccination, facemasks, and social distancing are unnecessary because they do not effectively protect people against COVID-19; on the other hand, they have their own harms*.” Male, unknown age.

Some people believed that there are commercial purposes behind the spread of SARS-CoV-2 to sell vaccines in the future.

“*This virus is human-made, and they created its vaccine before spreading it. They began this crime in order to force governments to purchase the vaccine. Who can guarantee that no unpredictable event would occur after the COVID-19 vaccine injection, and that we will not be forced to use another medication*?” Female, 51 years old.

“*The World health organization and pharmaceutical companies do not care about people’s health”, said another participant, “More patients means more money and profit.*” Female, 24 years old.

Some people refused the COVID-19 vaccine as they did not trust officials and governments.

#### 3.6.2. Lack of Knowledge about COVID-19 Vaccine

Within this theme, we found three subthemes. Some people did not trust the news about the COVID-19 vaccine, and others believed that officials were concealing the truth about the vaccine. According to one of the participants:

“*The COVID-19 vaccine surely has complications, but nobody will tell us*.” Female, 27 years old.

Some people mentioned that there is insufficient information about the COVID-19 vaccine to allow them to accept it. A group of people preferred to wait and see how other people’s vaccinations turned out before receiving the vaccine. One participant stated:

“*I will not use the COVID-19 vaccine unless a majority of people inject it and I make sure of its safety*.” Female, 47 years old.

Some people were skeptical of the COVID-19 vaccine because it had not passed the standard stages of drug approval. Therefore, people were unsure about its safety and efficacy, especially given the novel strategies to produce the vaccines. According to one of the participants:

“*Complications of COVID-19 vaccine are not fully determined, and we cannot claim that contracting COVID-19 is worse than the complications of the COVID-19 vaccine*.” Female, 23 years old.

Another participant stated,

“*I think one year is insufficient to evaluate the complications of a vaccine.” Long-term complications of COVID-19 vaccine should also be studied*.” Female, 26 years old.

#### 3.6.3. Lack of Trust in the COVID-19 Vaccine

We identified seven subthemes within this theme because certain people did not trust the COVID-19 vaccine for different reasons. Such people were skeptical of the COVID-19 vaccine while others, including those who encouraged them to receive the COVID-19 vaccine, had not been vaccinated. According to one of our participants,

“*People in the developed countries are refusing to receive the COVID-19 vaccine and do not accept it. Therefore, they try to put it to test on people in the developing countries*.” Female, 38 years old.

Some other people did not trust pharmaceutical companies and scientists’ assessments of the COVID-19 vaccine:

“*Pharmaceutical companies are liars*.” Male, 36 years old.

Some people believed the COVID-19 vaccines were unreliable because they did not trust the techniques used to produce them or the unidentified components of the COVID-19 vaccine. One of the participants said:

“*There is more likely that I will receive the COVID-19 vaccine if it is made of the attenuated virus; compared to the mRNA vaccines*.” Male, 25 years old.

“*I will not inject the COVID-19 vaccine because they will inject us with something else instead of vaccine*”, Female, 44 years old.

Some people believed that the COVID-19 vaccines were either ineffective or did not induce long-term immunity. According to one of them:

“*People who are afflicted with COVID-19 are only immune for four to six months, and there is a risk of reinfection. Then how can the COVID-19 vaccine be effective*?” Female, 46 years old.

“*COVID-19 has different effects on individuals with different genomes*”, said another participant. *“So, one type of vaccine will not be effective for all people, especially those that are manufactured overseas*.” Female, 42 years old.

Some people had issues with the COVID-19 vaccine itself because they were afraid of it, believed it was harmful or had previous unpleasant experiences with vaccination. Some people were worried about the potential long-term or short-term complications related to the COVID-19 vaccine:

“*COVID-19 vaccine is more harmful than COVID-19 itself*.” Male, 20 years old

“*Mortality rate and paralysis of people who received the COVID-19 vaccine make me worried*.” Female, 24 years old.

“*COVID-19 vaccine causes infertility and autism*.” Female, 35 years old.

“*I think that a vaccine that has been produced in a short time would have some unknown side effects. I am afraid of these side effects*.” Female, 22 years old.

Some participants have experienced side effects of other vaccines:

“*I contracted polio by using the oral polio vaccine because one country was running human trials on the polio vaccine, without informing people*.” Female, 58 years old.

Some people refused to receive the COVID-19 vaccine as they believed that there was little evidence about COVID-19 and the possibility of mutations in it.

“*Scientists do not have enough information about the COVID-19 and the process of its mutations. Therefore, they are unable to produce an effective vaccine*.” Female, 42 years old.

Some people believed that they would not be able to access safe and effective vaccines against COVID-19 due to sanctions, improper transition, and other factors.

#### 3.6.4. Personal Issues

We also categorized this theme into three subthemes. Some people refused to receive the COVID-19 vaccine because they feared it might endanger them due to their underlying conditions:

“*One of my family members has an underlying disease that even contracting an inactivated virus can be dangerous for him*.” Female, 42 years old.

Some other participants declined to use COVID-19 because of its high price or financial issues. According to one of them:

“*I do not have enough money to purchase the COVID-19 vaccine. We will die as a result of starvation, not because of COVID-19*.” Female, 35 years old.

Some other participants refused the COVID-19 vaccination as they were dealing with several problems in their life:

“*We have enough problems in our life.” one of the participants said. “COVID-19 is better than such life*.” Female, 30 years old.

#### 3.6.5. Feeling No Need for the COVID-19 Vaccine

We found four subthemes in this theme. Some participants claimed that they were resistant to COVID-19 and did not need a vaccine:

“*I think I have been infected with COVID-19, and I do not need the vaccine*.” Female, 40 years old.

Others suspected that COVID-19 was a mild disease that did not require vaccination.

“*Coronaviruses have existed for a long time*.” one of the participants said. “*COVID-19 has a 3% mortality rate, particularly in those with a weak immune system*.” Female, unknown age.

Some people rejected the COVID-19 vaccine as they believed in the efficacy of the alternative preventive strategies. One of the participants said:

“*I prefer not to receive the COVID-19 vaccine. If other people get vaccinated, they will become immune too*.”Male, 36 years old.

“*I prefer to continue social distancing and other protective measures*.” Female, 59 years old.

The presence of effective treatment against COVID-19 was cited by others as a justification for refusing to receive the COVID-19 vaccine:

“*I prefer to use Imam Kazim medicine that I have witnessed its effects*.” one participant said. “*All of my family could go through the COVID-19 with minimal symptoms because of this medicine*.” Female, 42 years old.

“*I have been infected with COVID-19 three times, but I have recovered using vitamin C and thyme instead of tea*.” Male, 24 years old.

Some individuals refused to receive the COVID-19 vaccine because they believed it would interfere with the natural course of the disease, and they believed being infected with COVID-19 was better than vaccination. According to one of the participants:

“*In my opinion, older adults should receive the vaccine, but others should be infected with COVID-19 and enhance their immune system to produce the antibodies*.” Female, 30 years old.

Another participant said:

“*A disease is a natural event, and our bodies and nature have their own ways to face diseases. There is no need for external solutions*.” Female, 45 years old.

#### 3.6.6. Responsibility

A group of participants declined to have the COVID-19 vaccine as they believed that high-risk populations, including elderly adults and those with underlying illnesses, should be the priority to receive the COVID-19 vaccine. One of the participants said:

“*I am not one of the high-risk people” one of the participants stated* “*I prefer that those who are at high risk for COVID-19 infection receive the vaccine*.” Female, 22 years old.

## 4. Discussion

In this large-scale study, we evaluated different aspects of Iranian people’s opinions about the COVID-19 vaccine. To the best of our knowledge, this is the first research to perform a comprehensive evaluation of Iranian people’s reasons to accept or reject the COVID-19 vaccine, which may have practical implications in general population vaccination plans, not only in Iran but also in other developing countries, especially those who share similar cultural backgrounds with Iran. In our study, 69% of participants expressed a desire to receive the COVID-19 vaccine. If we consider 100% protection after vaccination, it was estimated that vaccination of 60% to 72% of people will be sufficient to achieve herd immunity against COVID-19 [25]. Under these circumstances, if all of those who intend to receive the vaccine actually follow through with their intentions, the COVID-19 vaccination coverage may be adequate to attain herd immunity against COVID-19 in Iran.

However, a number of factors may exacerbate the situation. First, new mutations of SARS-CoV-2 may increase its transmissibility [26], requiring more people to be vaccinated in order to achieve herd immunity [25]. Second, vaccines do not have 100% efficacy, and this issue could become more troublesome in developing countries such as Iran, as providing highly successful vaccines such as BioNTech/Pfizer [27] may be challenging in these countries due to cost and storage requirements. A higher vaccination rate will be needed to establish herd immunity with less effective vaccines [25]. Therefore, even though 69% of participants desired to receive the COVID-19 vaccine, it would be insufficient to guarantee herd immunity in Iran. Therefore, there is a serious need for interventions, including educational programs and campaigns to raise the public’s awareness of the issue and increase the adoption of the COVID-19 vaccine in Iran.

The general population’s acceptance of the COVID-19 vaccine varies significantly across countries. Lazarus et al. conducted an international survey to evaluate the acceptability of the COVID-19 vaccine in various countries. In their study, 71.5% mentioned that they would somehow receive the COVID-19 vaccine, with Russian residents having the lowest acceptance rate (54.9%), and Chinese residents having the highest acceptance rate (88.6%) [28]. In a systematic review, Sallam compared the approval of the COVID-19 vaccine among various populations in different countries [12]. In his study, the variation in the public’s acceptance was more prominent than reported in the Lazarus et al. study, with 97% of Ecuadorians desiring to receive the COVID-19 vaccine, while only 23.6% of Kuwait’s general population did [12]. In the Middle East and North Africa (MENA) region, the acceptance rate of the COVID-19 vaccine among the general population ranges from 23.6% in Kuwait to 66% in Turkey [12]. Our findings indicate that Iranians may have a better acceptance of the COVID-19 vaccine compared to other countries in the region. Acceptance of the COVID-19 vaccine by Iranians (69%) is relatively similar to the average acceptance rate in Lazarus et al. study (71.5%) [28]. Lack of knowledge about the COVID-19 vaccine and a lack of transparency in the news media related to the COVID-19 vaccine were frequently mentioned by our participants as reasons for rejecting the vaccine, which may be applicable to other countries as an important issue. Communication of government and scientific resources to the general population, as well as providing them with accurate information about vaccines, their components, their mechanism of action, their side effects, and their efficacy may increase the general public’s willingness to obtain the COVID-19 vaccine [28,29]. Such coordination is crucial to enhance vaccine acceptability because modern techniques used to manufacture COVID-19 vaccine, such as the mRNA vaccine [30], are new and untested, and lack of education in this regard may be a barrier to their acceptability [31].

In the current study, we found that adults who refused to receive the COVID-19 vaccine were significantly older than those who intended to receive it. This finding is different from previous studies’ results, as older age was associated with more willingness to receive the COVID-19 vaccine in the majority of previous studies [32,33,34,35,36,37]. Alqudeimat et al. evaluated the acceptance of the COVID-19 vaccine among the Kuwait general population. Their findings were similar to ours as the COVID-19 vaccine was less accepted among adults who were 55 to 64 years of age, and they mentioned regional differences as a cause for the discrepancy between their findings and other studies [38]. Our findings are also in contrast with protective behavior models, which predict that individuals who are more vulnerable to disease will take protective steps more seriously [39]. There may be several explanations for such findings. First, we found that adults aged 40 or older had more negative attitudes toward the COVID-19 vaccine and were less confident about it. However, we did not find an independent association between age and attitude toward the COVID-19 vaccine and confidence in it in our regression models. Therefore, older adults’ rejection of the COVID-19 vaccine may be because of a higher prevalence of other factors associated with a more negative attitude toward the COVID-19 vaccine in this group. For example, older adults may be less educated or knowledgeable about the COVID-19 vaccine than younger adults. Additionally, they may have less access to smartphones and websites and follow the COVID-19 news from more available sources such as TV news, which we found is correlated with a more negative attitude toward the COVID-19 vaccine. Furthermore, some of the participants mentioned that they were afraid of COVID-19 vaccine side effects because of their underlying conditions, which may be another reason for their lower intention to receive the COVID-19 vaccine. Whatever the reason is, our results suggest that this group requires special attention and communication, and their concerns or questions about the COVID-19 vaccine should be addressed, as older adults may benefit the most from the COVID-19 vaccine considering that older age and underlying health conditions are risk factors for mortality due to COVID-19 [40].

We found that Iranians who had lost one of their first-degree relatives due to COVID-19 had less intention to receive the COVID-19 vaccine; however, this finding did not reach statistical significance. In a study on the French working-age population, Schwarzinger et al. reported that those who knew someone hospitalized due to COVID-19 had a higher intention to receive the COVID-19 vaccine [41], which contrasts with our findings. Typically, the perceived severity of a health condition is an important factor that motivates protective behaviors [42]. Although individuals who have lost a relative due to COVID-19 are more likely to understand COVID-19 severity, in our sample, it appears that other factors play a larger role in vaccination decision-making. This community of people may be representative of those, who believe in conspiracy theories behind the COVID-19 pandemic and its vaccination, trust in complementary medicine as an effective treatment for COVID-19, or are less educated and do not have enough information about COVID-19 protective measures and the COVID-19 vaccine. As a result, they may have less motivation to take protective measures against COVID-19 and may refuse vaccination. Latkin et al. also found that those who were more engaged in preventive measures such as facemasks and social distancing showed a greater desire to receive the COVID-19 vaccine [43]. This pattern can be exacerbated by a lack of knowledge and misinformation [44]. In Iran’s cultural settings, authorities, religious clerics, and health officials can play critical roles in countering misinformation about vaccination by using public media and education to provide people with reliable, accurate, and trustworthy information [45]. TV programs can be utilized for this purpose because Iranians who relied on TV news as their main source of COVID-19 information had a lower willingness to receive the COVID-19 vaccine and may benefit from such educational programs.

In our study, females accepted the COVID-19 vaccine at a lower rate than males (66.6% vs. 73.3%). This result was consistent across trials, as females in most countries have expressed a lower willingness to receive the COVID-19 vaccine [12,32,33,35,36,38,41,43]. The only exception was Indonesia, where females were more eager to obtain the COVID-19 vaccine than males; again, the disparities were not statistically important [46]. Additionally, we found that the female gender is independently associated with a more negative attitude toward the COVID-19 vaccine and more concerns about vaccines’ safety. There may be several explanations for such differences. First, males experience more severe forms of COVID-19 [47] and are more vulnerable to COVID-19 than females, which may explain why they are more likely to accept the COVID-19 vaccine. Second, males exhibit more risk-taking behavior than females [48], and females may have less intention to use the COVID-19 vaccine considering the rapid development and approval of the vaccines. According to previous studies, a considerable number of females were unsure whether to accept or reject the COVID-19 vaccine [49]. Addressing their concerns and questions about the COVID-19 vaccine, especially its safety, may increase their acceptance of the vaccine. Some of our participants rejected the vaccine due to a lack of enough information about the long-term side effects and a lack of stage four clinical trials. People’s concerns in this regard may be logical, and their acceptance of the COVID-19 vaccination may grow over time as more studies on the efficacy and side effects of the COVID-19 vaccines are conducted. On the other hand, misinformation had a major role in the participants’ rejection of the COVID-19 vaccine. This finding is similar to Lockyer et al., who found that misinformation is a source of confusion and mistrust among the general population [50]. They also stated that people trust council websites and local officials [50]. However, in our study, some people mentioned that they do not trust scientists, authorities, and official news websites, making communication with people in Iran more complicated. Several strategies are suggested to face misinformation and misconception about the COVID-19 vaccine. Although scientists, celebrities, and politicians can play important roles as role models for many people, and promoting educational campaigns via highly influential people may be beneficial [51], it may also be important to work with key stakeholders at the community level. Additionally, scientists can be a reliable source of knowledge for people and have a critical role in combating misinformation. Another effective strategy may be to make the information provided by scientists widely available via public and popular media [51].

Several qualitative studies were conducted on people’s opinions and beliefs regarding the COVID-19 vaccine. In a study in England, Lockyer et al. found a lack of trust in authorities, contradictory information, and misinformation on the COVID-19 vaccine as sources of distress and confusion among people. Concerns about COVID-19 vaccine safety also affected people’s desire to receive the COVID-19 vaccine [50]. In another study in India, people had concerns about the efficacy of the COVID-19 vaccine, its safety, and its side effects, especially in the long term [52]. Our findings are also in line with these studies, as lack of trust in authorities and healthcare professionals, misinformations, and concerns regarding the safety of the COVID-19 vaccine were factors leading to COVID-19 vaccine hesitancy among our participants. Misinformations on COVID-19 have been a serious challenge and a major source of distress among populations since the beginning of the COVID-19 pandemic [53]. These misinformations may negatively affect the countries’ strategies to control the COVID-19 outbreak. Therefore, monitoring these misinformations on different platforms, providing people with trusted sources of COVID-19 news, and addressing people’s concerns may be beneficial in combating these misinformation and increasing acceptance of the COVID-19 vaccine. COVID-19 vaccine price and access to the effective vaccine were concerns for both Iranian and Indian people [52], which may be the case in other developing countries, too, as people in these countries have less financial power compared to people in developed countries and might not be able to afford to pay for vaccine themselves [54]. Additionally, the economic impacts of the COVID-19 pandemic might reduce the governments of developing countries’ ability to supply free or low price vaccines for their people, especially considering the high price of some available vaccines [55,56]. Policymakers must consider this issue at an international level as the inability to sufficiently vaccinate people in developing countries might lead to challenges in the global control of the pandemic.

In our study, 35.9% of participants were males, 3.5% lived in rural areas, 4.4% were older than 60 years, and 3.4% had an educational level lower than a high school diploma. It is a limitation of our study that the demographic makeup of our sample diverged from the actual characteristics of Iranians, as a larger percentage of Iranians are males, older than 60, and live in rural areas [57,58]. These differences were largely unavoidable, as we conducted this study as an online survey.

Our study had several strengths. First, people in our study came from all of Iran’s provinces, which increases the generalizability of our findings. Second, we developed three questionnaires in our study that evaluate different factors affecting people’s decision to accept or reject the COVID-19 vaccine. These questionnaires can be used in future studies and may enable researchers to compare their findings using reliable questionnaires. Third, we used a mixed qualitative–quantitative method, which allowed us to identify groups who tend to oppose the COVID-19 vaccine and who may benefit the most from possible educational programs to raise awareness about the COVID-19 vaccine and improve people’s acceptance of the COVID-19 vaccine. In addition, in the qualitative part, we evaluated the participants’ reasons to accept or reject the COVID-19 vaccine, which may provide a better understanding of people’s concerns and the factors contributing to the approval of the COVID-19 vaccine, and future programs may focus on these factors to increase general population acceptance.

The major limitation of our research was the use of an online questionnaire since people of lower socioeconomic status might have limited access to social media, which was used for recruitment in this study. However, considering the current pandemic situation and our goal of including people from all Iranian provinces, the use of an online questionnaire was necessary. Second, the online questionnaire was also a limitation in the qualitative part of our study as we were unable to further explore people’s opinions, especially the controversial ones, and in some cases, people reported only vague comments, which we could not evaluate for the qualitative analysis. Third, 4.4% of respondents were 60 years or more, which may reduce the generalizability of our findings for this age group. Fourth, due to the non-random nature of sampling, the study population may differ from the general population, and our study may be subject to sampling bias. Fifth, as volunteer subjects were recruited for this study, it is also subject to volunteer response bias. Finally, our study findings may have been influenced by social desirability bias due to assessing attitudes that may be perceived to have a “good” or “bad” connotation.

## 5. Conclusions

The rate of intention to have the COVID-19 vaccine among Iranian people is similar to the international average; however, it is still below where we need to be. There are serious challenges to a vaccination program in Iran, such as older adults’ lower intention to receive the COVID-19 vaccine, which must be addressed in future planning. Furthermore, lack of accurate information and misinformation are major barriers to COVID-19 vaccine hesitancy in the Iranian population, emphasizing the importance of providing people with reliable and trustworthy news on this subject. Our findings can be a cornerstone for addressing people’s concerns regarding the COVID-19 vaccine in order to increase its acceptance rate among the general population in Iran. Additionally, these findings enhance our knowledge of the reasons leading to the acceptance or rejection of vaccines in emergencies, which can be useful for policymakers in similar emergent conditions in the future.

## Figures and Tables

**Table 1:** Demographic characteristics of participants and their history of COVID-19 infection.

Variable		Number (Percent)
Age	15–29 years	780 (40.5%)
	30–39 years	516 (26.8%)
	40–49 years	355 (18.4%)
	50–59 years	193 (10%)
	>60 years	84 (4.4%)
Gender	Female	1236 (64.1%)
	Male	692 (35.9%)
Marital Status	Single	870 (45.1%)
	Married	995 (51.6%)
	Divorced or the partner has died	63 (3.3%)
Place of Living	Rural	68 (3.5%)
	Urban	1860 (96.5%)
Educational Level	Less than high school diploma	66 (3.4%)
	High school diploma (11 years of education)	461 (23.9%)
	College degree	108 (5.6%
	Bachelor’s degree	740 (38.4%)
	Master’s degree	360 (18.7%)
	Doctorate or higher degree	193 (10%)
Occupation	Unemployed	105 (5.4%)
	Housewife	267 (13.8%)
	Student	394 (20.4%)
	Healthcare worker	157 (8.1%)
	Employee	917 (47.6%)
	Retired	88 (4.6%)
Salary	Do not receive a salary	578 (30%)
	Less than 20 million Rials	332 (17.2%)
	Between 20 to 40 million Rials	413 (21.4%)
	Between 40 to 60 million Rials	228 (14.9%)
	More than 60 million Rials	317 (16.4%)
Source of COVID-19 News	TV news	411 (21.3%)
	Websites	332 (17.2%)
	Social media	1032 (53.5%)
	Other	153 (7.9%)
Daily Time Spent Following COVID-19 News	Do not follow the news on most days	476 (24.7%)
	Less than one hour a day	1080 (56%)
	Between one to two hours a day	265 (13.7%)
	More than two hours a day	107 (5.5%)
History of COVID-19 Infection in the Participants or Their First-Degree Relatives	No	765 (39.7%)
	Yes	1163 (60.3%)
History of Hospitalization Due to COVID-19 in the Participants or Their First-Degree Relatives	No	1527 (79.2%)
	Yes	401 (20.8%)
Death of First-degree relatives due to COVID-19	No	1727 (89.6%)
	Yes	201 (10.4%)

**Table 2:** Participants’ attitudes, concerns, and confidence regarding the COVID-19 vaccine based on their basic characteristics.

		Attitude		Lack of Confidence		Vaccine Safety Concern	
		Mean (SD)	*p*-Value	Mean (SD)	*p*-Value	Mean (SD)	*p*-Value
Age group	15–29 years	10.2 (3.39)	0.104	39.52(10.87)	0.001	15.48 (4.97)	0.368
	30–39 years	9.92 (3.6)		40.49 (11.12)		15.71 (4.74)	
	40–49 years	9.58 (3.8)		42 (11.05)		16.02 (4.09)	
	50–59 years	9.67 (3.51)		42.12 (9.76)		16.39 (3.56)	
	>60 years	9.84 (3.52)		41.5 (10.05)		16.14 (4.48)	
Gender	Female	9.69 (3.6)	<0.001	40.97 (10.93)	0.05	16.02 (4.54)	0.05
	Male	10.4 (3.4)		39.89 (10.74)		15.3 (4.7)	
Marital status	Single	10.25 (3.39)	<0.001	39.21 (10.76)	<0.001	15.3 (4.89)	0.001
	Married	9.76 (3.63)		41.53 (10.8)		16.09 (4.35)	
	Divorced or the partner has died	8.53 (3.8)		44.53 (11.17)		16.9 (4.15)	
Area of residency	Rural	9.61 (4.04)	0.698	41.91 (11.06)	0.301	16.1 (4.91)	0.301
	Urban	9.95 (3.53)		40.53 (10.86)		15.75 (4.6)	
Educational level	Less than high school diploma	7.98 (3.71)	<0.001	45.46 (9.65)	<0.001	17.98 (3.93)	<0.001
	High school diploma (11 years of education)	9.52 (3.75)		41.67 (10.84)		16.19 (4.56)	
	College degree	9.77 (2.97)		42.25 (8.68)		16.9 (4.14)	
	Bachelor’s degree	9.84 (3.53)		41.02 (11.15)		15.73 (4.58)	
	Master’s degree	10.51 (3.43)		38.92 (10.71)		15.34 (4.83)	
	Doctorate or higher degree	11.03 (3.04)		36.79 (10.25)		14.23 (4.36)	
Occupation	Unemployed	9.42 (3.92)	<0.001	42.06 (12.69)	<0.001	16.2 (5.29)	<0.001
	Housewife	9.4 (3.48)		42.37 (10.26)		16.91 (3.77)	
	Student	10.33 (3.31)		38.43 (10.42)		15.2 (4.95)	
	Healthcare worker	10.48 (3.62)		38.85 (11.04)		14.58 (4.54)	
	Employee	9.94 (3.6)		40.95 (10.96)		15.78 (4.62)	
	Retired	9.59 (3.25)		42.29 (9.35)		16.13 (3.76)	
Salary	Do not receive a salary	9.75 (3.57)	0.023	40.59 (11.23)	0.004	16.02 (4.85)	0.012
	Less than 20 million Rials	9.86 (3.41)		41.21 (10.74)		16.08 (4.48)	
	Between 20 to 40 million Rials	10.42 (3.58)		40.74 (10.75)		15.56 (4.57)	
	Between 40 to 60 million Rials	9.72 (3.73)		41.65 (10.75)		15.76 (4.27)	
	More than 60 million Rials	10.46 (3.39)		38.72 (10.65)		15.22 (4.63)	
Source of COVID-19 news	TV news	8.94 (3.46)	<0.001	44.19 (947)	<0.001	17.1 (3.79)	<0.001
	Websites	10.26 (3.56)		40.11 (10.82)		15.38 (4.24)	
	Social media	10.41 (3.37)		38.83 (10.81)		15.26 (4.85)	
	Other	8.74 (4.07)		43.7 (11.93)		16.37 (4.97)	
Daily time spent following COVID-19 news	Do not follow the news on most days	8.6 (3.82)	<0.001	44.71 (11.1)	<0.001	16.98 (4.66)	<0.001
	Less than one hour a day	10.25 (3.34)		39.54 (10.36)		15.54 (4.46)	
	Between one to two hours a day	10.64 (3.43)		39.01 (10.52)		15.1 (4.42)	
	More than two hours a day	11.14 (2.96)		36.64 (10.89)		14.15 (5.26)	
History of COVID-19 infection in the participants or their first-degree relatives	No	10.01 (3.6)	0.268	40.51 (11.04)	0.67	15.66 (4.72)	0.67
	Yes	9.9 (3.51)		40.63 (10.76)		15.82 (4.54)	
History of hospitalization due to COVID-19 in the participants or their first-degree relatives	No	10.03 (3.54)	0.02	40.37 (10.86)	0.033	15.64 (4.62)	0.033
	Yes	9.61 (3.57)		41.4 (10.87)		16.22 (4.56)	
Death of first-degree relatives due to COVID-19	No	10.02 (3.5)	0.007	40.38 (10.79)	0.011	15.67 (4.6)	0.011
	Yes	9.24 (3.86)		42.35 (11.38)		16.52 (4.68)	

**Table 3:** Results of multiple linear regressions.

Dependent Variable	Independent Variables	Beta	*p*-Value
	Female gender	−0.531	0.001
COVID-19 Vaccine Attitude Questionnaire	Educational degree of less than a high school diploma	−1.33	0.002
	Educational degree higher than master’s	0.814	<0.001
	Websites and social media as the main source of COVID-19 news	1.213	<0.001
	Not following COVID-19 news during the day	−1.615	<0.001
	Loss of the first-degree relative due to COVID-19	−0.849	0.001
COVID-19 vaccine lack of confidence inventory	Single marital status	−1.636	0.002
	Educational degree higher than master’s	−3.212	<0.001
	Student	−2.132	0.001
	Websites and social media as the main source of COVID-19 news	−3.959	<0.001
	Not following COVID-19 news during the day	5.222	<0.001
	Loss of the first-degree relative due to COVID-19	2.032	0.008
Modified vaccine safety concern scale	Female gender	0.569	0.008
	Educational degree higher than master’s	−0.831	<0.001
	Healthcare worker	−0.987	0.01
	Using websites and social media as the main source of COVID-19 news	−1.397	<0.001
	Not following COVID-19 news during the day	1.42	<0.001

**Table 4:** Association between participants’ desire to receive the COVID-19 vaccine and basic characteristics, their attitude, confidence, and concerns about the COVID-19 vaccine.

Variables		Expect to Receive COVID-19 Vaccine		*p*-Value
		No	Yes	
Age (years)		35.97 (12.5)	34.4 (12.83)	0.001
Gender	Female	413 (33.4%)	823 (66.6%)	0.002
	Male	185 (26.7%)	507 (73.3%)	
Marital status	Single	240 (27.6%)	630 (72.4%)	0.011
	Married	335 (337%)	660 (66.3%)	
	Divorced or the partner has died	23 (36.5%)	40 (63.5%)	
Area of residency	Rural	21 (30.9%)	47 (69.1%)	1
	Urban	577 (31%)	1283 (69%)	
Educational level	Less than high school diploma	25 (37.9%)	41 (62.1%)	0.726
	High school diploma	146 (31.7%)	315 (68.3%)	
	College degree	33 (30.6%)	75 (69.4%)	
	Bachelor’s degree	234 (31.6%)	506 (68.4%)	
	Master’s degree	105 (29.2%)	255 (70.8%)	
	Doctorate or higher degree	55 (28.5%)	138 (71.5%)	
Occupation	Unemployed	37 (32.5%)	68 (64.8%)	0.032
	Housewife	82 (30.7%)	185 (69.3%)	
	Student	95 (24.1%)	299 (75.9%)	
	Healthcare worker	49 (31.2%)	108 (68.8%)	
	Employee	305 (33.3%)	612 (66.7%)	
	Retired	30 (34.1%)	58 (65.9%)	
Salary	Do not receive a salary	173 (29.9%)	405 (70.1%)	0.0.137
	Less than 20 million Rials	103 (31%)	229 (69%)	
	Between 20 to 40 million Rials	139 (33.7%)	274 (66.3%)	
	Between 40 to 60 million Rials	100 (34.7%)	188 (65.3%)	
	More than 60 million Rials	83 (26.2%)	234 (73.8%)	
Source of COVID-19 news	TV news	143 (34.8%)	268 (65.25)	0.004
	Websites	107 (32.2%)	225 (67.8%)	
	Social media	287 (27.8%)	745 (72.2%)	
	Other	61 (39.9%)	92 (60.1%)	
Daily time spent following COVID-19 news	Do not follow the news on most days	212 (44.5%)	264 (55.5%)	<0.001
	Less than one hour a day	308 (28.5%	772 (71.5%)	
	Between one to two hours a day	64 (24.2%)	201 (75.8%)	
	More than two hours a day	14 (13.1%)	93 (86.9%)	
History of COVID-19 infection in the participants or their first-degree relatives	No	248 (32.4%)	517 (67.6%)	0.291
	Yes	350 (30.1%)	813 (69.9%)	
History of hospitalization due to COVID-19 in the participants or their first-degree relatives	No	469 (30.7%)	1058 (69.3%)	0.585
	Yes	129 (32.2%)	272 (67.8%)	
Death of first-degree relatives due to COVID-19	No	528 (30.6%)	1199 (69.4%)	0.227
	Yes	70 (34.8%)	131 (65.2%)	
COVID-19 vaccine attitude questionnaire		6.92 (3.6)	12.17 (2.55)	<0.001
COVID-19 vaccine lack of confidence inventory		50.52 (8.54)	36.11 (8.62)	<0.001
Modified vaccine safety scale		18.51 (3.56)	14.53 (4.5)	<0.001

Values are reported as numbers (percentage), except for age, attitude, confidence, and safety concern scores, which are reported as mean (SD).

**Table 5:** Thematic analysis of participants’ reasons to accept or reject the COVID-19 vaccine.

Acceptance or Rejection	Theme	Subtheme	Code
Acceptant of the COVID-19 vaccine	Ending the COVID-19 pandemic	Controlling the pandemic	Controlling COVID-19 dissemination
			Reaching herd immunity
		Tough pandemic situation	Getting tired of quarantine
			Getting tired of taking health measures
			Getting tired of the current situation
		Returning to the pre-pandemic situation	Improvement of the situation
			To have social interactions
			Return to normality
			Return to job
	Responsibility	Social responsibility	Social duty
			Prevention from SARS-CoV-2 mutation
			Encouraging other people to receive the COVID-19 vaccine
		Protecting other people	Helping those who have a contraindication to receiving the COVID-19 vaccine
			Helping people who can not receive the COVID-19 vaccine
			Immunity of other people
			Prevention of the transmission of COVID-19 to others
			Protection of family members
			The underlying diseases of family members
		Helping HCWs and scientists	Reduction in the psychological burden on HCWs
			Help the HCWs
			Increasing the knowledge about the COVID-19 vaccine
	Becoming immune to COVID-19	Prevention of being infected	Prevention from being a carrier of SARS-CoV-2
			Immunity against the COVID-19
			Immunity of family
		Prevention of severe forms of COVID-19	Prevention from death due to COVID-19
			Prevention from the severe forms
			Prevention of the complications
		Susceptibleness to COVID-19	Having underlying diseases
			Occupational exposure
			Being infected by COVID-19 in the past
	Psychological well-being	Fear of contracting COVID-19	Concerns about family
			Fear of COVID-19
			Concerns about contracting COVID-19
		Psychological issues of COVID-19 pandemic	Ease of concerns in social life
			Alleviation of the psychological impacts of the COVID-19 pandemic
	Hopefulness about vaccine effectiveness	Cost-effectiveness	Possibility of the COVID-19 vaccine efficiency
			Harms of current situation compared to COVID-19 vaccine
			Higher odds of COVID-19 vaccine benefits
		Taking action against the COVID-19	Avoid being passive
			Taking a risk
			Lack of an alternative
	Trust in the COVID-19 vaccine	Current scientific evidence	Trust in scientific resources
			Trust in the experts
			Trust in the science
		Prior experiences with vaccination	History of vaccination during the life
			Prior successful vaccination programs
			Vaccination against COVID-19 by others
Reject to receive the COVID-19 vaccine	Unknown policies behind COVID-19 pandemic	Spreading the COVID-19 purposefully	Using COVID-19 vaccine to control the world
			Using COVID-19 vaccine to harm people
			Using COVID-19 to control the population growth
			Secret goals of producing COVID-19 vaccine
			COVID-19 being human-made
		Commercial purposes of spreading COVID-19	COVID-19 conspiracy to sell the vaccine
			Commercial purposes of producing COVID-19 vaccine
		Lack of trust in the current policies	COVID-19 being a political game
			Lack of trust in governments
			Lack of trust in the officials
	Lack of knowledge about COVID-19 vaccine	Untrustworthy news	Hiding the truth
			Rumors
			Unreliable news
		Lack of enough information on COVID-19 vaccine	Lack of enough data on COVID-19 vaccine
			Wait to see the vaccination results
		Lack of enough studies on COVID-19 vaccine	Undetermined vaccine complications
			Undetermined vaccine efficacy
			Lack of routine studies
			Novel vaccine components
	Lack of trust in COVID-19 vaccine	Other people’s practice toward COVID-19 vaccine	Other people not getting vaccinated
			Encouragers of COVID-19 vaccine are not vaccinated
		Lack of trust in scientific resources	Lack of trust in scientists
			Lack of trust in pharmaceutical companies
		Unreliability of COVID-19 vaccine	Lack of trust in techniques to produce COVID-19 vaccine
			Lack of trust in COVID-19 vaccine
			Undetermined components of COVID-19 vaccine
		Low quality of the COVID-19 vaccine	Ineffectiveness of COVID-19 vaccine
			The inability of vaccines to induce long-term immunity
		Issues with COVID-19 vaccine	COVID-19 vaccine hazards
			Fear of COVID-19 vaccine
			Previous experiences of vaccine complications
		Nature of SARS-CoV-2	SARS-CoV-2 mutations
			Lack of awareness about SARS-CoV-2
		Lack of access to effective vaccine	Lack of access to high-quality vaccine
			Uncertainty in the safe transition of COVID-19 vaccine
	Personal issues	Underlying conditions	Underling disease
			History of allergy
		Financial issues	Financial pressures
			The high price of the vaccine
		Problems in life	Tough situations in life
	Feeling no need for COVID-19 vaccine	Resistance to COVID-19	History of being infected with COVID-19
			Having a strong immune system
			Lack of underlying disease
		Nature of COVID-19	COVID-19 being a mild disease
		Using preventive strategies	Herd immunity with vaccination of other people
			Effectiveness of preventive strategies
			Not going out
		Possible treatments	Using effective medications
		Not interfering with the natural course of COVID-19	Being infected is better than vaccination
			Necessity of following the natural process
	Responsibility	-	Others being in priority

## Data Availability

The datasets used and/or analyzed during the current study are available from the corresponding author on reasonable request.

## Supplementary Materials

The following supporting information can be downloaded at: https://www.mdpi.com/article/10.3390/vaccines10050670/s1, Supplementary S1: Questionnaires used in the study; Supplementary S2: Mean participants’ scores of COVID-19 vaccine attitude questionnaire items; Supplementary S3: Mean participants’ scores of COVID-19 vaccine lack of confidence inventory items; Supplementary S4: Mean participants’ scores of modified vaccine safety scale items.
