# Supplementary material for: Acceptance or Rejection of the COVID-19 Vaccine: A Study on Iranian People’s Opinions toward the COVID-19 Vaccine
Source: Vaccines (Basel). 2022 Apr 23;10(5):670. doi: 10.3390/vaccines10050670 (PMC9143028; doi:10.3390/vaccines10050670)
Supplement: Supplementary file 1 [file vaccines-10-00670-s001.zip › Supplementary S1.pdf]

1. COVID-19 vaccine attitude questionnaire

	Agree (0)	Neither agree nor disagree (1)	Disagree (2)
If large populations get immune against COVID-19, few adverse reactions are acceptable. (R)			
There is not much scientific evidence for the safety of the COVID-19 vaccine.			
Instead of preventing, the COVID-19 vaccine causes the disease.			
The risks of getting a COVID-19 vaccine outweigh its benefits.			
The COVID-19 vaccine should never be given to the elderly.			
In general, getting COVID-19 is safer than getting vaccinated against it.			
What do you think about the COVID-19 vaccination in general? (R)			

2. COVID-19 vaccine – lack-of-confidence inventory

	1	2	3	4	5
COVID-19 vaccines have not been adequately tested for safety.					
Getting vaccinated against COVID-19 helps protect people who are unable to get vaccinated. (R)					
The COVID-19 vaccines can cause or worsen allergies.					
Improved living standards, not vaccination, can reduce the incidence of COVID-19.					
People should be able to decide whether or not to vaccinate against COVID-19.					
Pharmaceutical companies purposefully conceal information about the safety of COVID-19 vaccines.					
COVID-19 will be virtually eliminated, so vaccination is not needed.					
COVID-19 vaccines cause the disease they are supposed to prevent.					
The government conceals information about the safety of COVID-19 vaccines.					
Homeopathic medicines are an effective alternative to COVID-19 vaccines.					
COVID-19 vaccines introduce toxins into the body.					
The more people who get vaccinated, the greater the protection against COVID-19. (R)					
Building immunity by naturally fighting off COVID-19 is better protection than getting the vaccine.					
It is okay for people to be exempt from COVID-19 vaccination for moral or personal reasons.					
Pharmaceutical companies create ineffective COVID-19 vaccines for profit.					

(1) strongly disagree to *(5) strongly agree*

3. Modified vaccine safety concerns scale

	1	2	3	4	5
Mercury in vaccines can cause autism in newborns.					
Vaccines can cause diabetes.					
Vaccines can cause cancer.					
Vaccines can cause bovine spongiform encephalopathy (mad cow disease).					
Vaccines can cause sudden infant death syndrome.					
Vaccines can cause infertility.					

(1) strongly disagree to (5) strongly agree
