# Supplementary material for: Acceptance or Rejection of the COVID-19 Vaccine: A Study on Iranian People’s Opinions toward the COVID-19 Vaccine
Source: Vaccines (Basel). 2022 Apr 23;10(5):670. doi: 10.3390/vaccines10050670 (PMC9143028; doi:10.3390/vaccines10050670)
Supplement: Supplementary file 1 [file vaccines-10-00670-s001.zip › Supplementary S2.pdf]

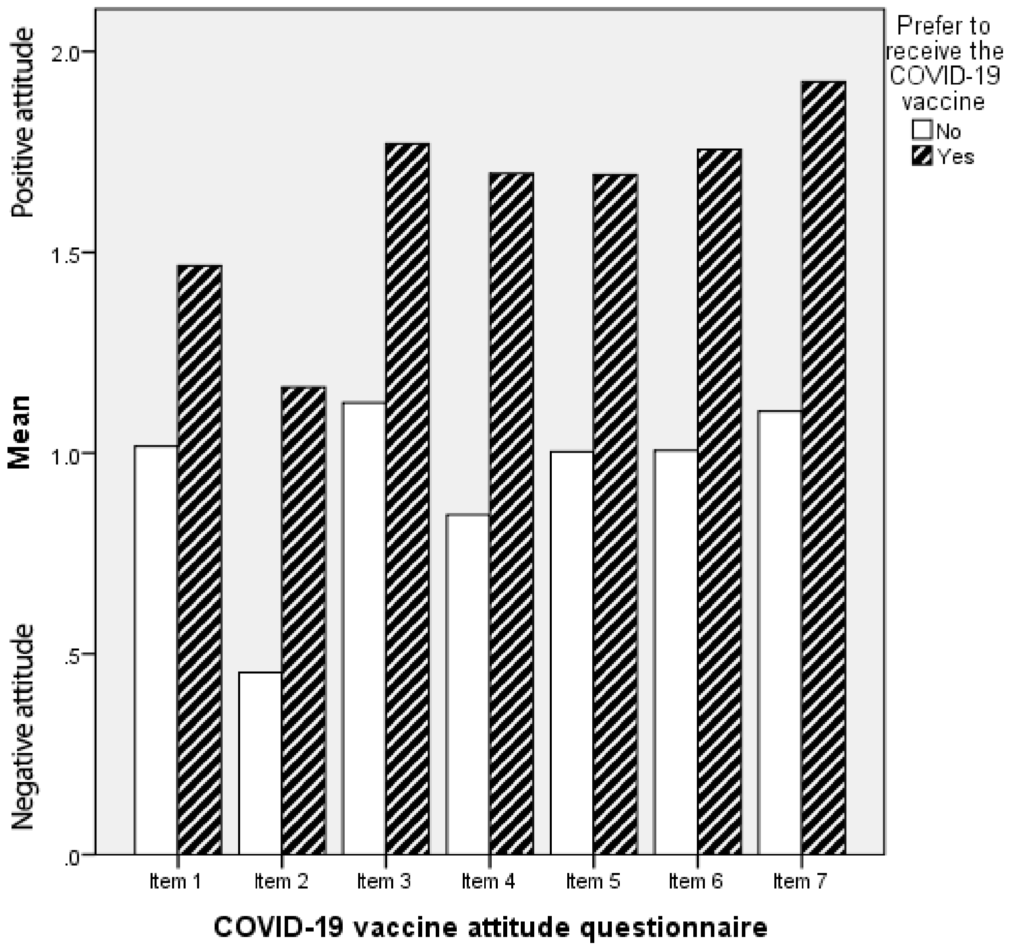

Figure S1- Mean participants' scores of COVID-19 vaccine attitude questionnaire items.

Items: 1.If large populations get immune against COVID-19, few adverse reactions are acceptable. 2.There is not much scientific evidence for the safety of the COVID-19 vaccine. 3.Instead of preventing, the COVID-19 vaccine causes the disease. 4.The risks of getting a COVID-19 vaccine outweigh its benefits. 5. The COVID-19 vaccine should never be given to the elderly. 6. In general, getting COVID-19 is safer than getting vaccinated against it. 7. What do you think about the COVID-19 vaccination in general?
