# Supplementary material for: Acceptance or Rejection of the COVID-19 Vaccine: A Study on Iranian People’s Opinions toward the COVID-19 Vaccine
Source: Vaccines (Basel). 2022 Apr 23;10(5):670. doi: 10.3390/vaccines10050670 (PMC9143028; doi:10.3390/vaccines10050670)
Supplement: Supplementary file 1 [file vaccines-10-00670-s001.zip › Supplementary S3.pdf]

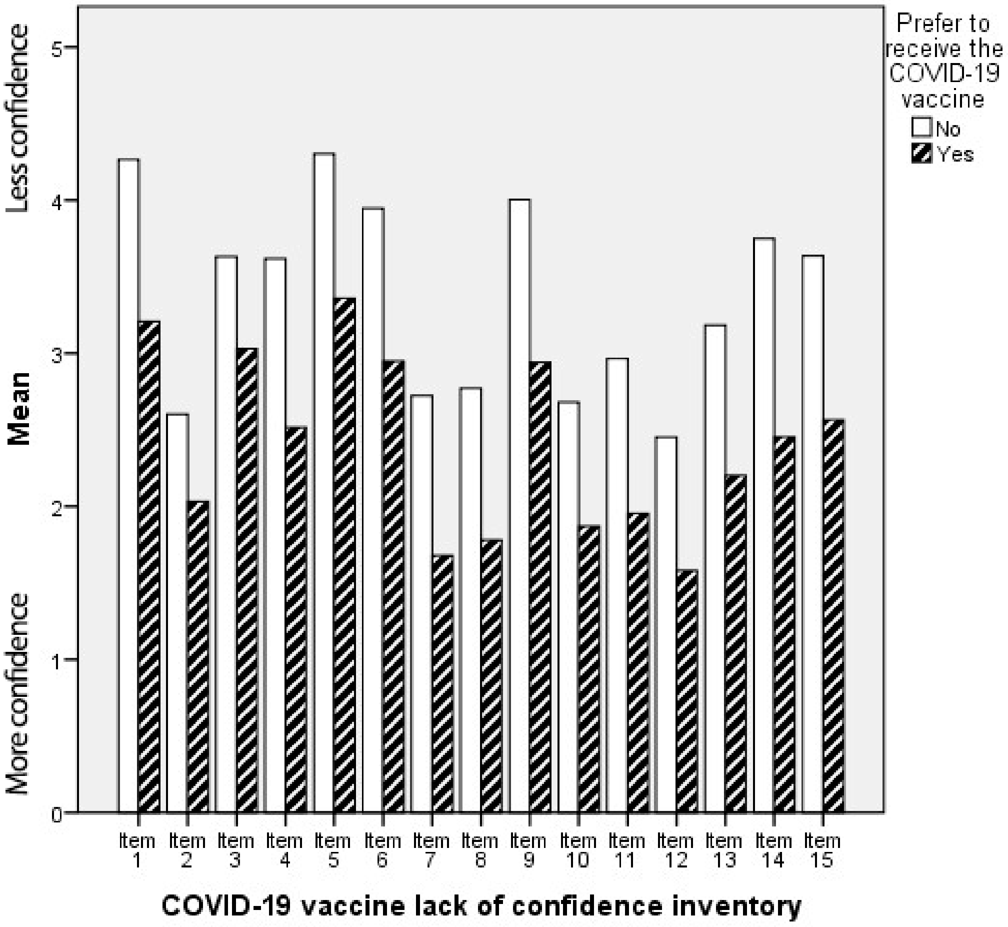

Figure S2- Mean participants' scores of COVID-19 vaccine lack of confidence inventory items.

Items: 1.COVID-19 vaccines have not been adequately tested for safety. 2.Getting vaccinated against COVID-19 helps protect people who are unable to get vaccinated. 3. The COVID-19 vaccines can cause or worsen allergies. 4. Improved living standards, not vaccination, can reduce the incidence of COVID-19.5. People should be able to decide whether or not to vaccinate against COVID-19. 6. Pharmaceutical companies purposefully conceal information about the safety of COVID-19 vaccines. 7. COVID-19 will be virtually eliminated, so vaccination is not needed. 8. COVID-19 vaccines cause the disease they are supposed to prevent. 9. The government conceals information about the safety of COVID-19 vaccines. 10. Homeopathic medicines are an effective alternative to COVID-19 vaccines. 11. COVID-19 vaccines introduce toxins into the body. 12. The more people who get vaccinated, the greater the protection against COVID-19. 13. Building immunity by naturally fighting off COVID-19

is better protection than getting the vaccine. 14. It is okay for people to be exempt from COVID-19 vaccination for moral or personal reasons. 15. Pharmaceutical companies create ineffective COVID-19 vaccines for profit.
