# Supplementary material for: Acceptance or Rejection of the COVID-19 Vaccine: A Study on Iranian People’s Opinions toward the COVID-19 Vaccine
Source: Vaccines (Basel). 2022 Apr 23;10(5):670. doi: 10.3390/vaccines10050670 (PMC9143028; doi:10.3390/vaccines10050670)
Supplement: Supplementary file 1 [file vaccines-10-00670-s001.zip › Supplementary S4.pdf]

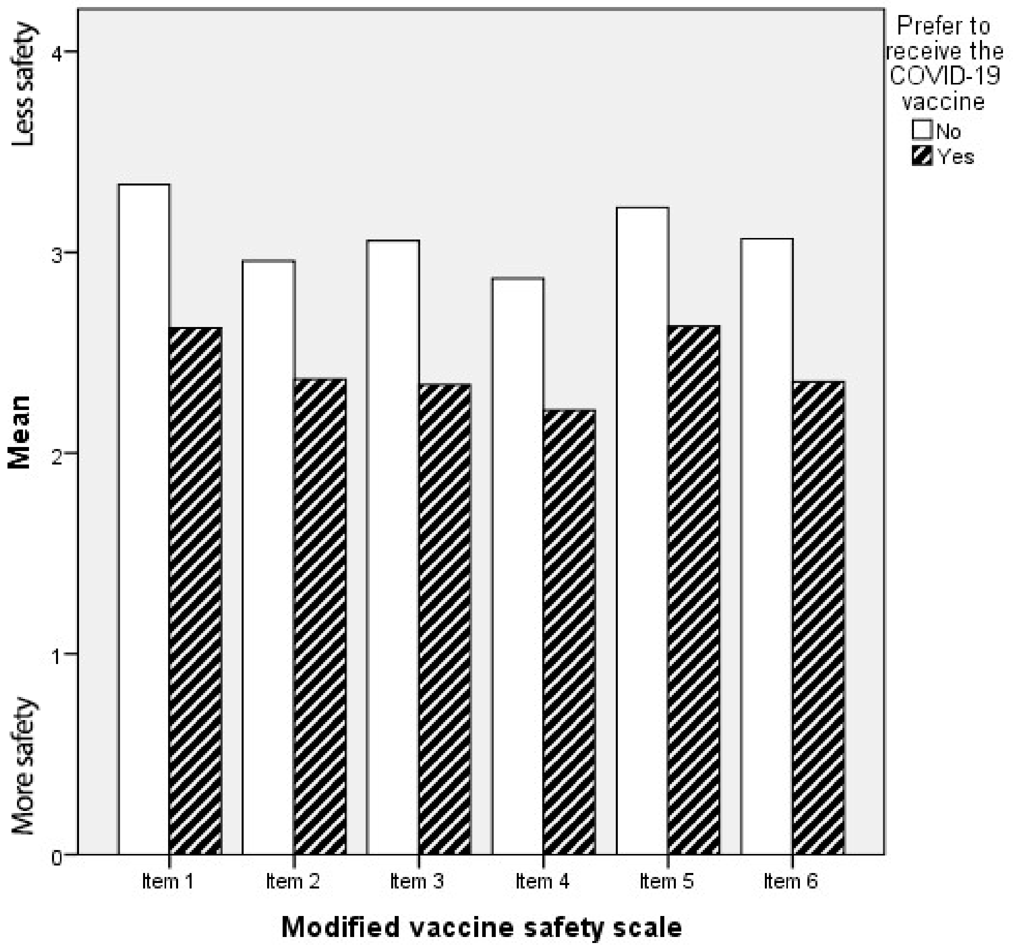

Figure S3- Mean participants' scores of modified vaccine safety scale items.

Items: 1. Mercury in vaccines can cause autism in newborns. 2. Vaccines can cause diabetes. 3. Vaccines can cause cancer. 4. Vaccines can cause bovine spongiform encephalopathy (mad cow disease). 5.Vaccines can cause sudden infant death syndrome. 6. Vaccines can cause infertility.
